# Evaluating the impact of caries prevention and management by caries risk assessment guidelines on clinical practice in a dental teaching hospital

**DOI:** 10.1186/s12903-016-0217-9

**Published:** 2016-05-26

**Authors:** Gillian H. M. Lee, Colman McGrath, Cynthia K. Y. Yiu

**Affiliations:** Paediatric Dentistry & Orthodontics, Faculty of Dentistry, University of Hong Kong, 2/F, Prince Philip Dental Hospital, 34 Hospital Road, Hong Kong SAR, China; Periodontology and Public Health, Faculty of Dentistry, University of Hong Kong, 3/F, Prince Philip Dental Hospital, 34 Hospital Road, Hong Kong SAR, China

**Keywords:** Guidelines, Guidelines implementation, Evaluation, Oral health, Children, Caries risk assessment, Dental caries, Prevention, ADAPTE, Delphi consensus

## Abstract

**Background:**

Clinical practice guidelines on ‘*Dental caries prevention and management by caries risk assessment for pre-school children in Hong Kong’* were developed using ADAPTE process and Delphi consensus technique. This study aimed to evaluate the feasibility of disseminating and implementing the guidelines, and to evaluate their effectiveness in changing clinical practice.

**Methods:**

The study was conducted in two phases, examining clinical records of pre-school aged patients being treated by non-academic clinical staff in the Paediatric Dentistry Clinic of a dental teaching hospital in Hong Kong. The clinical guidelines were introduced to the staff in a departmental seminar at the end of pre-intervention phase. Post-intervention phase began one month after the introduction of guidelines. Clinical records for three consecutive months were reviewed against standards and recommendations derived from the newly developed clinical guidelines in both phases. The results were assessed by Chi-square test, ANOVA and regression analyses.

**Results:**

A total of 237 and 147 clinical records were reviewed in pre-intervention and post-intervention phases, respectively. Guideline adherence percentage increased significantly on almost all aspects of the guidelines in the post-intervention phase (*P* < 0.05). There were a significant difference in the mean overall guideline adherence score (pre-intervention phase: $\overline{\mathrm{x}}$ = 14.86 ± 6.11; post-intervention phase: $\overline{\mathrm{x}}$ = 28.88 ± 8.75) and sub-domain adherence scores between the two phases (*P* < 0.001). The training grade of the clinicians was the factor associated with changes in evidence-based practice (*P* < 0.001).

**Conclusions:**

The developed guidelines were effective in translating evidence into best practice. The findings have implication for widespread implementation.

**Electronic supplementary material:**

The online version of this article (doi:10.1186/s12903-016-0217-9) contains supplementary material, which is available to authorized users.

## Background

There is a growing interest in evidence-based dentistry. Clinical practice guidelines are the key means to summarise and translate rapidly changing research evidence into practice and to assist with clinical decision making [1, 2]. Implementing guidelines in clinical practice can improve overall health service management, reduce variations in service delivery, improve the quality of care and ultimately the effectiveness of services [3, 4]. A Cochrane review has reported that the introduction of clinical practice guidelines can be effective in changing the process and outcome of care by professions allied to medicine [5]. However, evidence of change in the dental setting is limited.

The degree of adherence to guidelines in clinical practice following guideline implementation can vary considerably [6]. Potential barriers for guideline adherence relate to the social context – professional and patient attitudes, the organisational context – practice and resources available, and indeed the guidelines themselves – relevance and evidence [2, 7]. A number of approaches to implementing guidelines have been proposed including interactive seminars and educational meetings, multifaceted interventions, use of reminders and outreach educational visits [8, 9].

In Hong Kong, dental caries among pre-school children remains a concern; affecting one in two children and with over 90 % of untreated dental caries [10]. The condition remains similar over the past decade [11]. Previously, we have reported on wide variations in caries management approaches (treatment decision making) for pre-school children in Hong Kong [12]. Furthermore, we identified unfavourable attitudes to the provision of dental care to children among Hong Kong dentists [13]. To address these problems and in collaboration with the *Hong Kong Society of Paediatric Dentistry* (HKSPD), we developed clinical practice guidelines on ‘*Dental caries prevention and management by caries risk assessment for pre-school children in Hong Kong’* through the ADAPTE process and Delphi consensus technique among HKSPD members [14]. ADAPTE process is a comprehensive framework for guideline adaptation, while Delphi technique is a formal iterative structured process that aims to gather consensus of opinion, judgement or choice among a panel of experts. The Hong Kong guidelines on caries prevention and management by caries risk assessment comprise of consensus evidence-based recommendations on ‘caries diagnosis’, ‘caries risk assessment’, ‘preventive strategies for pre-school children at population level and for high risk individual’ and ‘restorative management strategies’. As university teaching hospitals are key to how future dentists practice evidence-based care, we aimed to evaluate the effectiveness of implementing the guidelines in terms of practice adherence in the management of pre-school children, pre- and post- guideline implementation in a dental teaching hospital.

## Methods

Clinical records of pre-school children (aged 5 years or younger) seen by the twelve non-academic clinical staff working within the Paediatric Dentistry Clinic at dental teaching hospital in Hong Kong were reviewed for a period of 3-months prior to guideline implementation. On average, each clinician would see around four to five patients in a treatment session. The patients visited the Clinic for all range of oral health care. There was no guidance or regulations on the prevention and management of dental caries for young children prior to the study. The clinical staff made their own treatment decision entirely based on their knowledge and experience.

A pro forma was developed to record practices relating to ‘caries risk assessment and caries diagnosis’ (16 aspects), ‘preventive strategies for high risk groups (including behaviour modification on dietary advice/oral hygiene instruction and prescription of preventive measures)’ (up to 39 aspects) and ‘restorative management strategies’ (11 aspects). These ‘aspects’ were related to the newly developed clinical guidelines on ‘*Dental caries prevention and management by caries risk assessment for pre-school children in Hong Kong’*. In addition, background information of the patients, such as gender, age and decayed, missing, and filled teeth (dmft) scores, and information of their corresponding dentists including gender and training grade were collected.

The developed guidelines were introduced by way of an interactive seminar involving non-academic clinical staff. A copy of the printed guidelines in form of booklets, pamphlets and electronic forms were disseminated to the clinicians. This approach was selected as this was the most common strategy to disseminate clinical guidelines [8, 9] and would be easily translatable to the widespread implementation of the guidelines among the dental practitioners in Hong Kong at a later time.

**Fig. 1:**
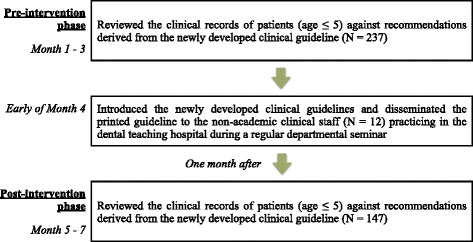
Design of the intervention in the study

All clinical records of pre-school children (aged 5 years or younger) seen by the twelve non-academic clinical staff for a period of 3 months, one month after the implementation of the guidelines, were reviewed and assessed using the standardised pro forma as described above. The clinical records were typed and digitally recorded in the hospital patients’ data system. The records were also kept in patients’ folders in print. The clinical staff were not aware of the review and assessment of their patients’ clinical records in both the pre- and post-intervention phases. The process of implementation and assessment is presented in Fig. 1.

Data were coded and analysed using IBM® SPSS® Statistics 20 (SPSS Inc., Chicago, IL, USA). Profile of patient’s characteristics were produced and compared. Variations in relation to individual aspects of the guidelines prior to and after intervention were compared employing Chi-square tests. In addition, the differences in the mean overall guideline adherence score and sub-domain adherence scores (‘caries risk assessment and caries diagnosis’, ‘preventive strategies for high risk groups’ and ‘restorative management strategies’) between the two phases were compared and analysed using Mann-Whitney U tests. Following on, a series of regression analyses (negative binomial) was conducted to identify operator and patient factors associated with changes in evidence-based practice (i.e., adherence to guidelines as documented on patients’ records). The level of statistical significance was set at α = 0.05.

The study was approved by Institutional Review Board of the University of Hong Kong/Hospital Authority Hong Kong West Cluster (HKU/HA HKW IRB, IRB reference number: UW14-278). All patients or parents/guardians of patients under 18 years old visiting the dental teaching hospital were consented to have the patients’ clinical records made available for teaching and research purposes. As such, parental consent to review the patients’ clinical records had been obtained among the patients involved in the study. All participants (the clinical staff) were consented to participate in the study and to publish the collected data. Data collected were stripped of personal identifiers.

## Results

The profile of patients is presented in Table 1. Clinical records of 237 patients (male: 138; female: 99), with a mean age of 4.29 (SD = 0.84) were reviewed in the pre-intervention phase. Post implementation of the guidelines, there were 147 patients (male: 76; female: 71), with a mean age of 4.55 (SD = 0.70). There was a significant difference in the age of the patients (*P* < 0.01) between the two phases. The mean dmft of those reviewed prior to the implementation was 9.63 (SD = 5.90) and was 8.94 (SD = 6.04) for those patients reviewed after the guideline implementation. The high dmft score indicated that majority of the patients (over 70 % in pre-intervention phase and 80 % in post-intervention phase) involved in the study had high caries experience.

**Table 1:** Patient characteristics and details of their corresponding operator in the study

Patient characteristics	Pre-intervention (*N* = 237)	Post-intervention (*N* = 147)	*P*-value
Gender			0.211
Male	138 (58.2 %)	76 (51.7 %)	
Female	99 (41.8 %)	71 (48.3 %)	
Age			0.002 * ^#^
Mean age in years	4.29 ± 0.84	4.55 ± 0.70	
< 2 years old	8 (3.4 %)	3 (2.0 %)	
3 years old	32 (13.5 %)	9 (6.1 %)	
4 years old	79 (33.3 %)	39 (26.5 %)	
5 years old	118 (49.8 %)	96 (65.3 %)	
dmft score			0.270 ^#^
Mean	9.63 ± 5.90	8.94 ± 6.04	
0	32 (13.5 %)	30 (20.4 %)	
1–5	31 (13.1 %)	13 (8.8 %)	
6–10	65 (27.4 %)	39 (26.5 %)	
11–15	64 (27.0 %)	44 (29.9 %)	
16–20	45 (19.0 %)	21 (14.3 %)	

Chi-square test; ^*#*^ Independent Sample T-test; * statistically significant (*P* ≤ 0.05)

The overall guideline adherence score and sub-domain adherence scores measuring the operators’ level of adherence to the guideline recommendations in the pre-intervention and post-intervention phases are shown in Table 2. The mean overall guideline adherence score in post-intervention phase was significantly higher (pre-intervention phase: $\overline{\mathrm{x}}$ = 14.86 ± 6.11; post-intervention phase: $\overline{\mathrm{x}}$ = 28.88 ± 8.75, (*P* < 0.001)). There were also significant differences in all the sub-domain adherence scores between the two phases as well (caries risk assessment and caries diagnosis adherence score: pre-intervention phase: $\overline{\mathrm{x}}$ = 3.48 ± 1.04, post-intervention phase: 4.87 ± 1.09, (*P* < 0.001); preventive strategies for high risk groups adherence score: pre-intervention phase: $\overline{\mathrm{x}}$ = 4.96 ± 3.66, post-intervention phase: $\overline{\mathrm{x}}$ = 11.48 ± 5.64, (*P* < 0.001); behaviour modification on dietary advice adherence score: pre-intervention phase: $\overline{\mathrm{x}}$ = 1.15 ± 1.60, post-intervention phase: $\overline{\mathrm{x}}$ = 3.59 ± 2.40, (*P* < 0.001); behaviour modification on oral hygiene instruction adherence score: pre-intervention phase: $\overline{\mathrm{x}}$ = 2.10 ± 1.45, post-intervention phase: $\overline{\mathrm{x}}$ = 6.28 ± 2.83, (*P* < 0.001); prescription of preventive measures adherence score: pre-intervention phase: $\overline{\mathrm{x}}$ = 1.71 ± 1.59, post-intervention phase: $\overline{\mathrm{x}}$ = 3.32 ± 1.45, (*P* < 0.001); restorative management strategies adherence score: pre-intervention phase: $\overline{\mathrm{x}}$ = 6.42 ± 2.70, post-intervention phase: $\overline{\mathrm{x}}$ = 10.82 ± 2.87, (*P* < 0.001)).

**Table 2:** Mean guideline adherence score and sub-domain adherence scores in the pre-intervention and post-intervention phase

	Pre-intervention (*N* = 237) Mean ± SD	Post-intervention (*N* = 147) Mean ± SD	*P*-value
Overall guideline adherence score	14.86 ± 6.11	28.88 ± 8.75	<0.001*
Caries risk assessment and caries diagnosis adherence score	3.48 ± 1.04	4.87 ± 1.09	<0.001*
Preventive strategies for high risk groups adherence score	4.96 ± 3.66	11.48 ± 5.64	<0.001*
Behaviour modification on dietary advice adherence score	1.15 ± 1.60	3.59 ± 2.40	<0.001*
Behaviour modification on oral hygiene instruction adherence score	2.10 ± 1.45	6.28 ± 2.83	<0.001*
Prescription of preventive measures adherence score	1.71 ± 1.59	3.32 ± 1.45	<0.001*
Restorative management strategies adherence score	6.42 ± 2.66	10.82 ± 2.87	<0.001*

*statistically significant (*P* ≤ 0.05), Mann-Whitney *U* test

The percentage of practice adherence to various aspects of the guideline recommendations are given as tables in the Additional file 1. A significant increase in the percentage of practice adherence in almost all aspects of guidelines was observed (*P* < 0.05). For individual aspects like ‘*interval for recalling/reviewing patient*’ and ‘*bitewing radiographs prescribed for caries diagnosis*’ under ‘*caries risk assessment and caries diagnosis*’; and ‘*provided glass ionomer under conventional restorative approach*’, ‘*provided glass ionomer for class II cavity*’ and ‘*provided stainless steel crown under conventional restorative approach*’ under ‘*restorative management strategies*’, the percentage of practice adherence was similar with no significant difference for these aspects between the two phases. There were no significant differences in the practice on ‘*recommended use of fluoride mouthrinse to caregiver*’, ‘*recommended use of antibacterials (chlorhexidine) to caregiver*’, and ‘*recommended use of probiotics*’ under ‘*prescription of preventive measures*’;

Findings of the overall negative binomial regression model identified that ‘training grade of the operators’ was associated with guideline adherence (*P* < 0.001), Table 3. Compared to the guideline adherence score of Junior Hospital Dental Officers (JHDOs) (first year graduates joining the training pathway), the expected log count of Year I and Year II post-graduate increased by 0.46 and 0.21 respectively, while decreased by 0.31 for the Year III post-graduate. The guideline adherent score of Year I and Year II post-graduates were 1.58 and 1.23 times higher than that of JHDOs respectively, while for Year III post-graduates, the score was 0.73 times that of the JHDOs.

**Table 3:** Negative binomial regression analysis predicting operator’s adherent to guidelines (overall guideline adherence score) (*N* = 384)

			95 % confidence interval			
Variable	B	Std. error	Lower bound	Upper bound	Exp(B)	*P*-value
(Intercept)	2.899	0.054	2.795	3.006	18.163	<0.001*
Training grade of the operators						
Post-graduate Training Year III	−0.311	0.153	−0.608	−0.004	0.733	0.042*
Post-graduate Training Year II	0.208	0.762	0.058	0.358	1.232	0.006*
Post-graduate Training Year I	0.456	0.102	0.257	0.660	1.578	<0.001*
Junior Hospital Dental Officer	0	–	–	–	1	–
(Negative binomial)	0.192	0.024	0.151	0.245		

$$ \mathbf{\mathcal{X}} $$ ^2^ (3) = 29.48, *P* < 0.001*

*statistically significant (*P* ≤ 0.05)

## Discussion

The present study was conducted in a dental teaching hospital involving a relatively large number of patients (*N* = 347), but a limited number of clinicians (*N* = 12) – involving junior hospital dental officers and residents (post-graduates) undergoing specialist training in paediatric dentistry. Clearly this has limitation in generalising the results to the wider practice in the community. Nonetheless, it does provide a useful pilot of the feasibility of implementing the developed guidelines, and to determine the effectiveness of implementing the guidelines in terms of clinical practice.

There was a decrease in the number of patients seen by the clinical staff in the post-intervention phase (a drop of 90 patients). The clinical staff had less clinical sessions because of public holidays during the three-month period of post-intervention phase. Therefore, they saw less patients. Since evaluation of the clinical practice of the staff prior to and after the guideline implementation should be the same (a fixed period of three consecutive months), the difference in the number of patients seen in the pre- and post- intervention phases had to be accepted. The significant difference in the age of patients seen in pre- and post-intervention phases was related to the difference in age distribution of the two phases. This occurrence was by chance. There was no effect of age difference on the changes in clinical practice of the staff, as shown by the result of the regression analysis.

In implementing guidelines, various multifaceted strategies have been considered to tailor implementation to the individual setting [2, 9, 15–22]. In this study, the implementation was by way of interactive seminars with discussion and dissemination of the published guidelines in print and electronic forms. This is the most common strategy to disseminate clinical guidelines to date [8, 9]. Moreover, this approach is translatable to the widespread implementation of the guidelines among the dental practitioners in Hong Kong at a later time. Continuing professional education lectures can be organised to introduce the newly developed guidelines to the general dental practitioners. Electronic and printed copies of the guidelines can also be mailed and distributed to all dental practitioners in Hong Kong easily.

There was a significant improvement in caries risk assessment and caries diagnosis with respect to the clinical guidelines, providing evidence of guideline adherence. The majority of the patients in both phases (over 70 %) can be considered at high risk of caries, based on their mean dmft scores. Prior to the implementation of the guidelines, the practice of a formal caries risk assessment was not documented in any chart. However, post intervention, approximately half of the cases had a formal documentation of caries risk assessment. There was no significant difference in terms of period of recall intervals documented pre and post intervention. This, however, reflects the already established practice of frequency recall in that the vast majority were prescribed to be reviewed within 6 months. In terms of the use of radiographs, there was a significant improvement in the reported practice for caries diagnosis. However, there was no significant difference in the use of bitewing radiographs, but at both phases, the practice was high and there was a significant improvement in the timing/frequency of radiographs taken based on caries risk status.

Oral health behaviour is key to oral health and the role of diet and hygiene is acknowledged [23]. In terms of behaviour modification, there were significant improvements in the reporting of dietary advice. Of note, prior to guidelines implementation, the majority of patients charts did not have evidence specific to diet based on caries risk status; whereas post intervention, this was uncommon but evident among one in five of patients records. There was also an observed improvement in documented oral hygiene instruction and specifically with respect to the use of fluoride toothpaste and frequency of brushing. Nonetheless, advice on post-brushing habit remained low, but was higher post guidelines implementation. A welcome finding was a considerable increase in providing self-management goals for oral hygiene practice.

In terms of prescription of preventive measures, there were significant improvements post interventions. For example, the practice of using topical fluorides by way of fluoride varnish dramatically increased in line with evidence of its effectiveness [24]. The recommendation of xylitol containing products, while improved post guideline implementation was not common, this in part may relate to the limited focus and evidence with respect of xylitol-contained products until relatively recently [25]. Of note, recommending preventive measures to providing advice to caregivers was not practiced. This may reflect clinicians’ perceived role to be limited to that of the child.

Regarding restorative management of carious primary teeth, there were significant improvements observed. The restorative management is likely to reflect phase of treatment and relate specifically to cases – thus in many cases, restorative approaches were not applicable. However, a welcome finding was the increase in the provision of preventive care in conjunction with restorative care.

Adherence to clinical guidelines is crucial to translate the recommendations into practice, but may vary depending upon the clinician’s routine practice [26]. Findings from the regression analyses identified variations in guidelines adherence, with greater adherence among clinicians who were at an earlier time of their training. Clinical practice approach might be easier to change when it was still not well established and may also be attributed to differences in attitudes to learning.

While improvements in evidence based practice was evidence after guideline implementation, there is still room for improvement in many aspects. Progressive improvements in adherence to guidelines have been reported [27], and it would be of value to consider outcomes in the longer term.

## Conclusions

It was feasible to implement the evidence-based clinical practice guidelines on ‘*Dental caries prevention and management by caries risk assessment for pre-school children in Hong Kong*’ in the dental hospital setting. This pilot study proved useful in informing the implementation process and was effective at improving evidence-based practice. Impact of guideline adherence in terms of clinical outcomes is warranted. Ultimately, in time, the widespread implementation and evaluation of guidelines is important among clinicians, patients and caregivers.

## Abbreviations

dmft, decayed, missing, and filled teeth; HKSPD, Hong Kong Society of Paediatric Dentistry; JHDOs, junior hospital dental officers.

## Additional file

Additional file 1: Table S1. Percentage of practice adherence to Guidelines in the pre-intervention and post-intervention phase on caries risk assessment and caries diagnosis; **Table S2.** Percentage of practice adherence to Guidelines in the pre-intervention and post-intervention phase on behaviour modification (dietary advice); **Table S3.** Percentage of practice adherence to Guidelines in the pre-intervention and post-intervention phase on behaviour modification (oral hygiene instruction); **Table S4.** Percentage of practice adherence to Guidelines in the pre-intervention and post-intervention phase on prescription of preventive measures; **Table S5.** Percentage of practice adherence to Guidelines in the pre-intervention and post-intervention phase on restorative management of carious primary teeth. (DOCX 49 kb)
