# Supplementary material for: Evaluating the impact of caries prevention and management by caries risk assessment guidelines on clinical practice in a dental teaching hospital
Source: BMC Oral Health. 2016 May 26;16:58. doi: 10.1186/s12903-016-0217-9 (PMC4881058; doi:10.1186/s12903-016-0217-9)
Supplement: Additional file 1: Table S1. — Percentage of practice adherence to Guidelines in the pre-intervention and post-intervention phase on caries risk assessment and caries diagnosis; Table S2. Percentage of practice adherence to Guidelines in the pre-intervention and post-intervention phase on behaviour modification (dietary advice); Table S3. Percentage of practice adherence to Guidelines in the pre-intervention and post-intervention phase on behaviour modification (oral hygiene instruction); Table S4. Percentage of practice adherence to Guidelines in the pre-intervention and post-intervention phase on prescription of preventive measures; Table S5. Percentage of practice adherence to Guidelines in the pre-intervention and post-intervention phase on restorative management of carious primary teeth. (DOCX 49 kb) [file 12903_2016_217_MOESM1_ESM.docx]

Additional file 1

| **Table S1. Percentage of practice adherence to Guidelines in the pre-intervention and post-intervention phase on caries risk assessment and caries diagnosis** | | | | | |
| --- | --- | --- | --- | --- | --- |
|  | | **Pre-intervention (%)**  (N = 237) | **Post-intervention (%)**  (N = 147) | 𝓧***^2^*** | ***P* - value** |
| Formal caries risk assessment recorded | |  |  | 130.85 | <0.001* |
|  | Yes | 0 (0%) | 67 (45.6%) |  |  |
|  | No | 237 (100%) | 80 (54.4%) |  |  |
| Re-assess / update patient’s caries risk status based on risk, and not exceeding 12 months | |  |  | 263.93 | <0.001* |
|  | Yes | 0 (0%) | 4 (2.7%) |  |  |
|  | No | 237 (100%) | 143 (97.3%) |  |  |
| Recall / review patient based on caries risk | |  |  | 7.84 | 0.005* |
|  | Yes | 148 (62.4%) | 112 (76.2%) |  |  |
|  | No | 89 (37.6%) | 35 (23.8%) |  |  |
| Interval for recalling/reviewing patient | |  |  | 5.24 | 0.263 |
|  | At 3 to 4 months | 56 (23.6%) | 34 (23.1%) |  |  |
|  | At 6 months | 104 (43.9%) | 78 (53.1%) |  |  |
|  | At 9 months or more | 77 (32.5%) | 35 (23.8%) |  |  |
| Radiographs prescribed for caries diagnosis | |  |  | 13.35 | <0.001* |
|  | Yes | 193 (81.4%) | 139 (94.6%) |  |  |
|  | No | 44 (18.6%) | 8 (5.4%) |  |  |
| Bitewing radiographs prescribed for caries diagnosis | |  |  | 1.05 | 0.306 |
|  | Yes | 171 (72.2%) | 113 (76.9%) |  |  |
|  | No | 66 (27.8%) | 34 (23.1%) |  |  |
| Timing of radiographic examination done based on caries risk status | |  |  | 114.84 | <0.001* |
|  | Yes | 49 (20.7%) | 112 (76.2%) |  |  |
|  | No | 188 (79.3%) | 35 (23.85) |  |  |
| * statistically significant (*P*≤0.05), Chi-square test | | | | | |

| **Table S2. Percentage of practice adherence to Guidelines in the pre-intervention and post-intervention phase on behaviour modification (dietary advice)** | | | | | |
| --- | --- | --- | --- | --- | --- |
|  | | **Pre-intervention (%)**  (N = 237) | **Post-intervention (%)**  (N = 147) | 𝓧***^2^*** | ***P* - value** |
| Specific dietary advice based on patient’s caries risk | |  |  | 106.18 | <0.001* |
|  | Yes | 17 (7.2%) | 75 (51.0%) |  |  |
|  | Yes, but not recorded in details | 75 (31.6%) | 42 (28.6%) |  |  |
|  | No | 145 (61.2%) | 30 (20.4%) |  |  |
| Advised reducing consumption of sugary food/drinks | |  |  | 87.70 | <0.001* |
|  | Yes | 69 (29.1%) | 115 (78.2%) |  |  |
|  | No | 168 (70.9%) | 32 (21.8%) |  |  |
| Advised confining consumption of sugary food/drinks to meal times | |  |  | 88.22 | <0.001* |
|  | Yes | 30 (12.7%) | 85 (57.8%) |  |  |
|  | No | 207 (87.3%) | 62 (42.2%) |  |  |
| Advised avoid sleeping/napping with bottle/feeder cup | |  |  | 16.39 | <0.001* |
|  | Yes | 31 (13.1%) | 44 (29.9%) |  |  |
|  | No | 206 (86.9%) | 103 (70.1%) |  |  |
| Advised avoid putting sugary drinks into a bottle | |  |  | 15.15 | <0.001* |
|  | Yes | 22 (9.3%) | 35 (23.8%) |  |  |
|  | No | 215 (90.7%) | 112 (76.2%) |  |  |
| Advised having tooth-friendly snacks | |  |  | 71.99 | <0.001* |
|  | Yes | 12 (5.1%) | 58 (39.5%) |  |  |
|  | No | 225 (94.9%) | 89 (60.5%) |  |  |
| Provided self-management goals on dietary habits | |  |  | 117.29 | <0.001* |
|  | Yes | 9 (3.8%) | 70 (47.6%) |  |  |
|  | Yes, but not recorded in details | 82 (34.6%) | 46 (31.3%) |  |  |
|  | No | 146 (61.6%) | 31 (21.1%) |  |  |
| * statistically significant (*P*≤0.05), Chi-square test | | | | | |

| **Table S3. Percentage of practice adherence to Guidelines in the pre-intervention and post-intervention phase on behaviour modification (oral hygiene instruction)** | | | | | |
| --- | --- | --- | --- | --- | --- |
|  | | **Pre-intervention (%)**  (N = 237) | **Post-intervention (%)**  (N = 147) | 𝓧***^2^*** | ***P* - value** |
| Specific oral hygiene instruction given based on patient’s caries risk | |  |  | 178.90 | <0.001* |
|  | Yes | 6 (2.5%) | 94 (63.9%) |  |  |
|  | Yes, but not recorded in details | 181 (76.4%) | 46 (31.3%) |  |  |
|  | No | 50 (21.1%) | 7 (4.8%) |  |  |
| Advised brushing under parental supervision | |  |  | 36.14 | <0.001* |
|  | Yes | 129 (54.4%) | 124 (84.4%) |  |  |
|  | No | 108 (45.6%) | 23 (15.6%) |  |  |
| Advised using small head toothbrush | |  |  | 64.29 | <0.001* |
|  | Yes | 1 (0.4%) | 38 (25.9%) |  |  |
|  | No | 236 (99.6%) | 109 (74.1%) |  |  |
| Advised using fluoride toothpaste | |  |  | 185.91 | <0.001* |
|  | Yes | 26 (11.0%) | 118 (80.3%) |  |  |
|  | No | 211 (89.0%) | 29 (19.7%) |  |  |
| Advised on the amount of toothpaste | |  |  | 136.35 | <0.001* |
|  | Smear or pea-sized based on age | 3 (1.3%) | 74 (50.3%) |  |  |
|  | Not to use | 0 (0%) | 0 (0%) |  |  |
|  | None | 234 (98.7%) | 73 (49.7%) |  |  |
| Advised on the fluoride content of toothpaste | |  |  | 100.87 | <0.001* |
|  | ≤ 500 ppm / 1000 ppm | 19 (8.0%) | 79 (53.7%) |  |  |
|  | ≥ 1000 ppm | 1 (0.4%) | 1 (0.7%) |  |  |
|  | None | 217 (91.6%) | 67 (45.6%) |  |  |
| Advised on frequency of toothbrushing | |  |  | 77.86 | <0.001* |
|  | x 2 or more | 115 (48.5%) | 135 (91.9%) |  |  |
|  | None | 122 (51.5%) | 12 (8.2%) |  |  |
| Advised on timing of toothbrushing | |  |  | 184.72 | <0.001* |
|  | Yes | 8 (3.4%) | 99 (67.3%) |  |  |
|  | No | 229 (96.6%) | 48 (32.7%) |  |  |
| Advised on post-brushing habit | |  |  | 23.66 | <0.001* |
|  | Yes | 4 (1.7%) | 21 (14.3%) |  |  |
|  | No | 233 (98.3%) | 126 (85.7%) |  |  |
| Provided self-management goals on oral hygiene practice | |  |  | 174.34 | <0.001* |
|  | Yes | 5 (2.1%) | 90 (61.2%) |  |  |
|  | Yes, but not recorded in details | 167 (70.5%) | 50 (34.0%) |  |  |
|  | No | 65 (27.4%) | 7 (4.8%) |  |  |
| * statistically significant (*P*≤0.05), Chi-square test | | | | | |

| **Table S4. Percentage of practice adherence to Guidelines in the pre-intervention and post-intervention phase on prescription of preventive measures** | | | | | |
| --- | --- | --- | --- | --- | --- |
|  | | **Pre-intervention (%)**  (N = 237) | **Post-intervention (%)**  (N = 147) | 𝓧***^2^*** | ***P* - value** |
| Applied topical fluoride based on caries risk | |  |  | 67.90 | <0.001* |
|  | Yes | 88 (37.1%) | 118 (80.3%) |  |  |
|  | No | 149 (62.9%) | 29 (19.7%) |  |  |
| Type of topical fluoride used | |  |  | 58.79 | <0.001* |
|  | Fluoride varnish | 96 (40.5%) | 118 (80.3%) |  |  |
|  | Fluoride gel | 4 (1.7%) | 0 (0%) |  |  |
|  | Did not apply any topical fluoride | 137 (57.8%) | 29 (19.7%) |  |  |
| Frequency of topical fluoride application | |  |  | 67.61 | <0.001* |
|  | 3-4 months / 6 months | 85 (35.9%) | 116 (78.9%) |  |  |
|  | Yearly | 15 (6.3%) | 2 (1.4%) |  |  |
|  | Did not apply any topical fluoride | 137 (57.8%) | 29 (19.7%) |  |  |
| Prescribed remineralising product (CPP-ACP) based on caries risk | |  |  | 33.37 | <0.001* |
|  | Yes | 88 (37.1%) | 55 (37.4%) |  |  |
|  | No | 149 (62.9%) | 73 (49.7%) |  |  |
|  | Not applicable | 0 (0%) | 19 (12.9%) |  |  |
| Prescribed fissure sealant based on caries risk | |  |  | 131.53 | <0.001* |
|  | Yes | 70 (29.5%) | 37 (25.2%) |  |  |
|  | No | 157 (66.2%) | 32 (21.8%) |  |  |
|  | Not applicable | 10 (4.2%) | 78 (53.1%) |  |  |
| Recommended use of fluoride mouthrinse to caregiver | |  |  | 2.08 | 0.149 |
|  | Yes | 0 (0%) | 0 (0%) |  |  |
|  | No | 235 (99.2%) | 143 (97.3%) |  |  |
|  | Not applicable | 2 (0.8%) | 4 (2.7%) |  |  |
| Recommended xylitol containing products | |  |  | 11.51 | 0.009* |
|  | Yes, recommended wipe for child | 0 (0%) | 3 (2.0%) |  |  |
|  | Yes, recommended chewing gum for caregiver | 0 (0%) | 4 (2.7%) |  |  |
|  | Yes, recommended for both child and caregiver | 2 (0.8%) | 1 (0.7%) |  |  |
|  | No | 235 (99.2%) | 139 (94.6%) |  |  |
| Recommended use of antibacterials (CHX) to caregiver | |  |  | 19.97 | <0.001* |
|  | Yes | 0 (0%) | 0 (0%) |  |  |
|  | No | 237 (100.0%) | 135 (91.8%) |  |  |
|  | Not applicable | 0 (0%) | 12 (8.2%) |  |  |
| Recommended use of probiotics | |  |  | 13.28 | <0.001* |
|  | Yes | 0 (0%) | 0 (0%) |  |  |
|  | No | 236 (99.6%) | 137 (93.2%) |  |  |
|  | Not applicable | 1 (0.4%) | 10 (6.8%) |  |  |
| Provided self-management goals on dental visits | |  |  | 181.87 | <0.001* |
|  | Yes | 3 (1.3%) | 91 (61.9%) |  |  |
|  | Yes, but not recorded in details | 227 (95.8%) | 52 (35.4%) |  |  |
|  | No | 7 (3.0%) | 4 (2.7%) |  |  |
| * statistically significant (*P*≤0.05), Chi-square test | | | | | |

| **Table S5. Percentage of practice adherence to Guidelines in the pre-intervention and post-intervention phase on restorative management of carious primary teeth** | | | | | |
| --- | --- | --- | --- | --- | --- |
|  | | **Pre-intervention (%)**  (N = 237) | **Post-intervention (%)**  (N = 147) | 𝓧***^2^*** | ***P* - value** |
| Provided restorative treatment in conjunction with preventive treatment | |  |  | 19.21 | <0.001* |
|  | Yes | 86 (36.3%) | 87 (59.2%) |  |  |
|  | No | 151 (63.7%) | 60 (40.8%) |  |  |
| Provided treatment to teeth with caries progressing into dentine only | |  |  | 73.61 | <0.001* |
|  | Yes, only preventive / restorative | 98 (41.4%) | 52 (35.4%) |  |  |
|  | Yes, both preventive and restorative treatment | 86 (36.3%) | 82 (55.8%) |  |  |
|  | None | 53 (22.4%) | 13 (8.8%) |  |  |
| Performed indirect pulp capping technique where appropriate | |  |  | 12.80 | 0.002* |
|  | Yes | 5 (2.1%) | 1 (0.7%) |  |  |
|  | No | 33 (13.9%) | 5 (3.4%) |  |  |
|  | Not applicable | 199 (84.0%) | 141 (95.9%) |  |  |
| Prepared carious cavity with atraumatic restorative technique where appropriate | |  |  | 33.59 | <0.001* |
|  | Yes | 66 (27.8%) | 49 (33.3%) |  |  |
|  | No | 57 (24.1%) | 3 (2.0%) |  |  |
|  | Not applicable | 114 (48.1%) | 95 (64.6%) |  |  |
| Provided types / frequency of caries diagnostic method based on patient’s age/caries risk | |  |  | 99.31 | <0.001* |
|  | Yes, for oral examination only | 179 (75.5%) | 42 (28.6%) |  |  |
|  | Yes, for radiographic examination only | 1 (0.4%) | 0 (0%) |  |  |
|  | Yes, for both oral and radiographic examination | 47 (19.8%) | 104 (70.7%) |  |  |
|  | No | 10 (4.3%) | 1 (0.7%) |  |  |
| Followed protocol on restorative care based on patient’s age/caries risk/co-operativeness | |  |  | 5.07 | 0.024* |
|  | Yes | 229 (96.6%) | 147 (100%) |  |  |
|  | No | 8 (3.4%) | 0 (0%) |  |  |
| Provided glass ionomer under atraumatic restorative technique | |  |  | 7.69 | 0.021* |
|  | Yes | 67 (28.3%) | 47 (32.0%) |  |  |
|  | No | 81 (34.2%) | 31 (21.1%) |  |  |
|  | Not applicable | 89 (37.6%) | 69 (46.9%) |  |  |
|  | |  |  |  |  |
| Provided glass ionomer under conventional restorative approach | |  |  | 5.14 | 0.077 |
|  | Yes | 27 (11.4%) | 21 (14.3%) |  |  |
|  | No | 120 (50.6%) | 57 (38.8%) |  |  |
|  | Not applicable | 90 (38.0%) | 69 (46.9%) |  |  |
| Provided glass ionomer for Class II cavity | |  |  | 2.42 | 0.120 |
|  | Yes | 0 (0%) | 0 (0%) |  |  |
|  | No | 148 (62.4%) | 80 (54.4%) |  |  |
|  | Not applicable | 89 (37.6%) | 67 (45.6%) |  |  |
| Provided composite restoration under conventional restorative approach | |  |  | 6.09 | 0.048* |
|  | Yes | 119 (50.2%) | 55 (37.4%) |  |  |
|  | No | 33 (13.9%) | 24 (16.3%) |  |  |
|  | Not applicable | 85 (35.9%) | 68 (46.3%) |  |  |
| Provided stainless steel crown under conventional restorative approach | |  |  | 5.46 | 0.065 |
|  | Yes | 97 (40.9%) | 43 (29.3%) |  |  |
|  | No | 50 (21.1%) | 35 (23.8%) |  |  |
|  | Not applicable | 90 (38.0%) | 69 (46.9%) |  |  |
| * statistically significant (*P*≤0.05), Chi-square test | | | | | |
